# Improving Handover of Patients in Critical Care following Cardiac Surgery: an audit and successful implementation of an electronic quality improvement tool

**DOI:** 10.1186/1749-8090-10-S1-A122

**Published:** 2015-12-16

**Authors:** Lucy Radevsky, Ashok Kar, Neville Hutchinson, Andrew Hill, Ishtiaq Ahmed

**Affiliations:** 1 Department of Cardiac Anaesthesia, Royal Sussex County Hospital, Eastern Rd, Brighton, BN2 5BE, UK; 2 Department of Cardiac Surgery, Royal Sussex County Hospital, Eastern Rd, Brighton, BN2 5BE, UK

## Background/Introduction

Improving Handover of Patients in Critical Care following Cardiac Surgery: an audit and successful implementation of an electronic quality improvement tool

## Aims/Objectives

Following cardiac surgery, professional responsibility and accountability for patients is transferred from the operating theatre to critical care. Optimising 'handover' is imperative and we sought to improve safety, quality and effectiveness of this within our unit.

## Method

A closed loop audit for all post-operative patients (Aug 2014 - Nov 2014) was performed to assess the handover process including personnel, environment and documentation on the electronic patient record (Metavision).

Prior to the first cycle, relevant stakeholders were consulted - anaesthetists, cardiac surgeons and nurses. A subsequent survey determined 'gold standard criteria' for handover before undertaking the second cycle.

A new electronic tool was created on Metavision to implement a change where specific anaesthetic and surgical representatives could document their plans including parameters and post-operative instructions.

## Results

Our audit showed objective improvement in presence of team members, especially surgeons; more frequent 'sterile cockpit' environment; significantly better documentation to guide the critical care MDT.

## Discussion/Conclusion

We demonstrated improvements in handover by following a system that utilizes the F1 racing model in the critical care setting. Moreover, a novel electronic tab dedicated for patient handover has significantly improved documentation. With ongoing challenges of maintaining continuity of care, we suggest other units adopt our strategy.

